# Medicine

**Published:** 1911-03

**Authors:** R. Shingleton Smith


### Progress of the Medical Sciences.

### MEDICINE.

**Tuberculin in pulmonary tuberculosis.**—The crusade against consumption is making great progress everywhere : the general facts as to the ravages of the disease and the possibility of cure are now well known. The question now is not whether anything more can be done, but what is the best method of procedure. Dr. Mearns Fraser[1] remarks that so widespread have been the misery, poverty and death caused by consumption, that public

[1] *Clin. J.*, 1910–11, xxxvii. 149.

opinion all over the country is unanimous as to the urgency of the need for taking some action towards the prevention and cure of the disease. He comments largely on the cost of sanatoria, and on the large number of cases for which provision should be made. He considers that the amount of sanatorium accommodation that can reasonably be provided can only benefit a very small proportion of the persons suffering from consumption. He criticises the action of sanatoria from the various points of view—as curative agencies, for purposes of isolation, and as educational agencies—and he argues that the cost of the education is excessive. He therefore maintains that the results so far obtained in this country, as also in Germany, do not warrant the amount of money that is expended upon them, so far as the working classes are concerned. He believes that a better method is available, and he recommends the Council of the Borough of Portsmouth to adopt the system of tuberculin dispensaries, which has of late been so strongly advocated by Dr. Camac Wilkinson, and which is being carried on at the Tuberculin Dispensary in Kennington Road, London. Other dispensaries have also been established at Aldershot, Street (Somerset) and Irvine (Ayrshire). Dr. Fraser finally advises his council to establish such a dispensary at a cost of about £600 per annum, and he thinks it inadvisable at present to provide a sanatorium for the borough. He anticipates the following great advantages from this method : " That it does not interfere to any appreciable extent with a man's means of livelihood, for it does not entail, as does sanatorium treatment, maintenance for some months in a sanatorium. I have seen patients receiving treatment during a course of several months who were able to follow their occupations nearly all the time. The advantage of a treatment to a working man which does not necessitate his giving up his work and leaving his family unprovided for for months is too obvious to need enlarging upon. An essential to the success of any treatment for consumption is that the disease should be attacked before it has progressed to its later stages. This applies as much to tuberculin as to sanatorium treatment, and herein is a great practical point in favour of tuberculin dispensaries. I have pointed out before that it is the money difficulty which prevents the working classes from availing themselves of sanatorium treatment in the early—that is to say, the curable—stage of the disease. There is, however, no such obstacle in the way of securing dispensary treatment, the cost of which is comparatively trifling. It is only reasonable to assume, therefore, that large numbers of the working classes, who would otherwise only have consented to go into a sanatorium when they had become unfitted for work, would, if such an institution were available, present themselves for treatment at a tuberculin dispensary whilst yet in the early stage of the disease. And it is reasonably certain that if tuberculin treatment is as successful as it is claimed to be the majority of these early cases would be permanently cured without ever having had to give up their work. The advantages from an economic point of view, the saving that would be effected to sickness assurance societies, friendly societies and boards of guardians, if such a result could be obtained, are so obvious as to need no comment."[1]

An article on "Tuberculin and Dispensaries" sums up the question.[2] It recalls the rush to Berlin when Koch first stated his belief that his original tuberculin had the power of annihilating the activity of tubercle in the living body. The reaction that followed the gradual discovery that his tuberculin was not a reliable or even safe remedy was exceedingly disappointing, so that the treatment by injection was almost entirely given up by the great majority of the profession. However, it was never entirely discarded : numerous modifications by various workers have been from time to time used largely by their enthusiastic advocates; and at the present time the use of one form or another of tuberculin has become established, more especially in Germany, and the results which are daily accumulating must convince everyone, except the incurable sceptic, that the use of tuberculin is becoming the rule of the level-headed practitioner, rather than the practice of the enthusiastic faddist.

The work of Bandelier and Roepke, translated by Morland,[3] has greatly conduced to our increased knowledge of the uses of tuberculin, both as regards diagnosis and treatment. The much-debated question of the use of tuberculin in out-patient practice is summed up strongly in favour of its adoption, and it would appear to be already in far greater use in Germany than with us. It appears probable, therefore, that in the near future the method of treating individuals with tuberculosis by progressive doses of tuberculin will receive a more or less extensive trial. At present medical opinion is in a state of suspense on many points.

The question as to what form of tuberculin to administer in any case is still open to differences of opinion. Hubert Kehl[4] uses a combination of Koch's old tuberculin and new tuberculin bacillary emulsion.

A new preparation called Tebean has given good results in the hands of L. Steffen.[5] This preparation has been evolved by Professor Levy.

[1] *Morning Post*, December 24th, 1910. [2] *Brit. M. J.*, 1911, i. 265.

[3] *Tuberculin in Diagnosis and Treatment*, 1909.

[4] *Wien. med. Klin.*, No. 36, 1910.

[5] *München med. Wchnschr.*, 1910, lvii. 838.

A tuberculinum purum, described by Gabrilowitch (Washington Congress, 1908), in rapidly increasing doses gave excellent results. Dr. F. A. Deal [1] states that owing to the work of the past decade tuberculin now stands on a well-founded basis. He considers that the avoidance of reaction is now the rule, and he finds that the tuberculinum purum, properly used, gives no reaction of any significance even in advanced cases.

Many of the older modifications have had a long-extended trial with uncertain results. The tuberculocidin and antiphthisin of Klebs [2] were at one time much vaunted, especially by Karl von Ruck, [3] who at first suspected that Professor Klebs had not only removed the dangerous properties from the tuberculin, but that he had removed its remedial principles as well. However, he was afterwards so convinced of the utility of tuberculin that he states, "I can abide my time, for the profession must ultimately arrive at the truth."

Trudeau and Baldwin found that the effects of larger doses of antiphthisin were similar in all respects to those produced by small doses of tuberculin, and they believed that the preparation made by Klebs is practically a highly diluted tuberculin, and its effects on animals the same ; but neither tuberculocidin nor antiphthisin had any curative influence over experimental tuberculosis in the guinea-pig, neither had they any germicidal power over tubercle bacilli in vitro. Various other modifications have been designed by Béraneck, Denys, Krauss and Koch. These are all described in Bandelier and Roepke, as also is Spengler's bovine tuberculin, from which so much is now expected.

A good general summing up of theory and practice in the treatment of pulmonary tuberculosis has recently been given by W. Cecil Bosanquet. [4] He favours the desire to increase resistance by the use of tuberculins, and thinks we may start with such a dose as $\frac{1}{10000}$ mg. of T. R., working up gradually to $\frac{1}{500}$ mg. We should just fail to give a recognisable reaction in temperature in apyretic cases, we should not use it in febrile cases, and he is very sceptical as to its value by the mouth. However, he maintains that there is no specific remedy or universal mode of treatment. "Neither tuberculin, nor rest, nor work, nor mountain air, nor a sea voyage, nor a sanatorium is an infallible remedy." We have to study the individual patient, his circumstances and his idiosyncrasies ; then, armed with this knowledge, to try to raise his resistance to the bacillus and its poisons.

It has not yet been forgotten that after the announcement

[1] *Med. Rec.*, 1910, lxxviii. 952.
[2] *Med. Rec.*, 1895, xlviii. 871. [3] *Med. Rec.*, 1892, xli. 685.
[4] *Brit. M. J.*, 1911, i. 124.

by Koch of the result of his investigations,[1] and of the enthusiasm which was everywhere aroused, it was very soon discovered by Virchow[2] that various pernicious results of tuberculin were of common occurrence, that acute hyperæmias of brain and hemorrhagic infiltrations of lungs and lymph-glands, the development of fresh tubercular foci and laryngeal swelling of phlegmonous character, gave rise to his cautionary warning in the use of a drug which could produce such results.

Hence it is easy to understand the psychology of hesitation to adopt a treatment which has given such results.[3] P. K. Pel apologises for a reserve in the presence of a remedy still on its trial. There is much to dampen enthusiasm, and to keep us on the watch because of the many by-effects, such as headache, fever, insomnia, loss of appetite and weight, acceleration of pulse and general depression, the chance of acute exacerbations and complications, and of an individual hyper-susceptibility to tuberculin, which have often been observed. Our cliniques, hospitals and sanatoria have not yet established its efficacy upon unassailable evidence. Hence the proposals of Dr. Camac Wilkinson,[4] who argues for what is called the massive tuberculin treatment, and raises great hopes in proportion to the boldness of his methods, are not greeted by the profession with cordial acclamation, inasmuch as the method is held to be one of "kill or cure," and there is little collateral observation from other quarters to support his views. He advises the systematic use of tuberculin in test doses, and states that he had not found a single case of pleurisy which did not react to tuberculin. He considers the method of diagnosis by the tuberculin test to be absolutely reliable, and that the early treatment of tuberculosis by means of tuberculin is a real step forward in prophylaxis, of far greater efficacy than sanatorium methods have been. But it has been pointed out from work done at the Phipps Dispensary, Baltimore,[5] that tuberculin tolerance is not synonymous with tuberculous immunity, and that under large doses the disease may break out in other organs. It is futile to expect that patients with rapidly progressive disease should react favourably; they must be offering some resistance before we can hope that tuberculin will stimulate such resistance.

There appears to be some ground for the contention that the tuberculin test has produced a very exaggerated estimate

[1] *Brit. M. J.*, 1890, ii.; 1891, i. 125.

[2] *Berl. klin. Wchnschr.*, xxviii. 49, 1891.

[3] *Berl. klin. Wchnschr.*, 1909, xlvi. 1717.

[4] *Treatment of Consumption*, Macmillan, 1908; *Practitioner*, 1910, lxxxiv. 145.

[5] *Johns Hopkins Hosp. Bull.*, 1909, xx. 248.

of the proportion of the population affected. Dr. J. Edward Squire [1] points out that some 80 per cent. will react, but many never present any indications of this disease, and are no danger to others. Tuberculosis carriers, not suspected of tubercle bacilli, may or may not be in fairly good health, and the question of infection from this standpoint is a real danger. Childhood appears to be the time when most infections occur. It is stated by Pottinger [2] that nearly all are infected before the fourteenth year, and that a large proportion gain immunity from their own infection, and immunity against re-infection. He concludes that :—

1. Treatment with tuberculin for five or six months will immunise a large percentage of incipient cases.

2. That in the later stages tuberculin gives increased immunity.

3. With immunity so obtained the disease becomes more chronic.

4. That complications may be treated by its use.

5. That immunity so obtained is much greater than by the ordinary hygienic measures.

The dispensary system is now claimed to mark the dawn of a new era in our fight against tubercle bacilli. It was begun in Edinburgh in 1887 by Dr. Philip, at the Victoria Dispensary for Consumptives, adopted also by Calmette at Lisle in 1901, then with bewildering rapidity all over the continent, but only recently in England, at Paddington in 1909. In Germany the system, started in 1904, had extended so rapidly, that in 1909 there were 858 institutions at work.

These dispensaries should co-ordinate all the agencies of the district, should aid in diagnosis and treatment either with or without tuberculin, should be the means for securing early diagnosis, should provide for the supervision of the patients and their families, and should give social help as well as aiming at the discovery of contacts.

There is not yet any general dispensary service in this country, but the advancement of thought is in their favour, and the day is coming, as it has come in Germany, France, America and Canada.

In our own district we have the object-lesson given us by Dr. Hilda Clark at Street, who has organised a philanthropic dispensary scheme under which from 70 to 80 patients are being treated with tuberculin by the progressive dose method, subsidiary treatment being used when necessary for individual cases.

Dr. Thurnam, Nordrach-on-Mendip, a pioneer in this work,

[1] *Proc. Roy. Soc. Med.*, 1910, iv., Epidem. Sect., p. 27.

[2] *Med. Rec.*, 1910, lxxvii. 1,042.

remarks "that the ideal tuberculin dispensary will be worked in connection with a sanatorium for the after-treatment of patients who have received their first doses therein. I find every case demands the utmost care and individual research. The dosage, the times of increasing the doses, and the amount of the increases, have to be most carefully thought out."

The ideal scheme should involve the following essentials :—

(*a*) Compulsory notification.

(*b*) The sanatorium for early and curable cases.

(*c*) A working colony for convalescents.

(*d*) A dispensary in the centre of the city or district.

(*e*) A hospital for the isolation of advanced cases, and their treatment.

(*f*) A home for the hopelessly incurable.

R. SHINGLETON SMITH.

